# Associations between Trace Elements and Cognitive Decline: An Exploratory 5-Year Follow-Up Study of an Elderly Cohort

**DOI:** 10.3390/ijerph17176051

**Published:** 2020-08-20

**Authors:** Bianca Gerardo, Marina Cabral Pinto, Joana Nogueira, Paula Pinto, Agostinho Almeida, Edgar Pinto, Paula Marinho-Reis, Luísa Diniz, Paula I. Moreira, Mário R. Simões, Sandra Freitas

**Affiliations:** 1 Center for Research in Neuropsychology and Cognitive and Behavioral Intervention (CINEICC), Faculty of Psychology and Educational Sciences (FPCEUC), Univ Coimbra, 3000-115 Coimbra, Portugal; joananogueira.f@gmail.com (J.N.); simoesmr@fpce.uc.pt (M.R.S.); sandrafreitas0209@gmail.com (S.F.); 2 Psychological Assessment and Psychometrics Laboratory (PsyAssessmentLab), Faculty of Psychology and Educational Sciences (FPCEUC), Univ Coimbra, 3000-115 Coimbra, Portugal; paulapinto663@hotmail.com; 3 Geobiotec Research Centre, Department of Geosciences, University of Aveiro, 3810-193 Aveiro, Portugal; marinacp@ua.pt (M.C.P.); pmarinho@dct.uminho.pt (P.M.-R.); 4 LAQV/REQUIMTE, Laboratory of Applied Chemistry, Faculty of Pharmacy, University of Porto, 4050-313 Porto, Portugal; aalmeida@ff.up.pt (A.A.); ecp@ess.ipp.pt (E.P.); luisa2diniz@gmail.com (L.D.); 5 Department of Environmental Health, School of Health, P.Porto, CISA/Research Center in Environment and Health, 4200-072 Porto, Portugal; 6 Departamento de Ciências da Terra, Instituto de Ciências da Terra, Polo da Universidade do Minho, Campus de Gualtar, 4710-057 Braga, Portugal; 7 Center for Neuroscience and Cell Biology (CNC), University of Coimbra, 3004-504 Coimbra, Portugal; pimoreira@fmed.uc.pt; 8 Institute of Physiology, Faculty of Medicine, University of Coimbra, 3000-548 Coimbra, Portugal

**Keywords:** cognitive decline, longitudinal study, risk for dementia, trace elements, nickel, selenium, human tissues, industrial area, mining area

## Abstract

Trace elements (TE) homeostasis is crucial in normal brain functioning. Although imbalances have the potential to exacerbate events leading neurodegenerative diseases, few studies have directly addressed the eventual relationships between TE levels in the human body and future cognitive status. The present study aimed to assess how different TE body-levels relate to cognitive decline. This exploratory research included a study-group (RES) of 20 elderly individuals living in two Portuguese geographical areas of interest (Estarreja; Mértola), as well as a 20 subjects neuropsychological control-group (CTR). Participants were neuropsychologically assessed through the Mini Mental State Examination (MMSE) and the Montreal Cognitive Assessment (MoCA) and the RES group was biomonitored for TE through fingernail analysis. After 5 years, the cognitive assessments were repeated. Analyses of the RES neuropsychological data showed an average decrease of 6.5 and 5.27 points in MMSE and MoCA, respectively, but TE contents in fingernails were generally within the referenced values for non-exposed individuals. Higher levels of Nickel and Selenium significantly predicted lesser cognitive decline within 5 years. Such preliminary results evidence an association between higher contents of these TE and higher cognitive scores at follow-up, suggesting their contribution to the maintenance of cognitive abilities. Future expansion of the present study is needed in order to comprehensively assess the potential benefits of these TE.

## 1. Introduction

The rapid aging of the world’s population is a current phenomenon that imposes great social and economic challenges. It is estimated that by 2050, there will be 1.5 billion people with ages above 65 years worldwide, corresponding to 16% of the population [1]. It is further foreseen that this proportion will be much higher in specific countries, such as Italy, Portugal, Greece, Japan, and Korea, where it will exceed the one-third mark [2]. Specifically, in Portugal, the estimations predict a ratio of 464 elderly (≥65 years old) per 100 young individuals by 2060, the quadruple of the ratio registered in 2001 [3]. Such aging rebounds in a growing number of people living with dementia—a condition characterized by the gradual loss of cognitive and functional capacities that interferes with the performance and execution of daily-living activities—as its prevalence/incidence increases sharply with age (from 2.3% among 65–69 years old individuals to 42% among elderly aged 90 or older) [2]. With a forecast of 41 million cases across OECD countries by 2050 [2], dementia is currently a major public health priority [4].

Strict genetic etiology is rare in dementia, being the vast majority of cases sporadic in origin [5,6,7]. Among elderly people, Alzheimer’s disease (AD) is the most frequent form of the condition and is mainly expressed by an accentuated loss of memory, as well as by the progressive decline of other cognitive and functional capacities. At the cellular level, AD pathology is characterized by neurofibrillary tangles (abnormal accumulations of hyperphosphorylated tau protein) and senile plaques (aggregations of amyloid-β protein) [8]. Several factors have been reported as contributors to the development of sporadic AD—besides aging, genetics is another well-established risk factor, with the presence of the APOE ε4 allele being the strongest genetic elicitor of increased risk for the disease [9]. Meanwhile, environmental exposure to toxic metals, to certain chemical compounds (such as pesticides and industrial chemicals) and to air pollutants have also been proposed as a risk factor [5,7,10].

Although not all environmental contaminants/toxins have been tested in regards to their effects on the central nervous system, it is hypothesized that there may be a risk for older adults to develop AD (and other neurodegenerative diseases such as Parkinson’s disease) that is associated with the neurologic impairments resulting from such environmental exposures [10,11,12,13]. Trace elements homeostasis is crucial in normal brain functioning, and disturbances may exacerbate AD-associated events [14,15]. Amongst trace metals, the relationship between AD and aluminum (Al) seems to be the best understood, with larger environmental studies, epidemiological studies and case-control evidence supporting an association between high levels of exposure to Al and the risk for AD [11,16]. For instance, this association has been suggested by studies reporting high rates of AD among older adults exposed to high concentrations of the metal [17], as well as by evidence of higher blood Al levels among AD patients (e.g., [18,19,20]). Copper (Cu) is another widely investigated element. Post-mortem case-control studies report a significant decrease of Cu in brain tissues of AD patients [21,22], particularly in region such as the hippocampus, amygdala [23], frontal cortex [24], cerebellum, motor and sensory cortex, cingulate gyrus, temporal gyrus, and entorhinal cortex [25]. In turn, research on iron (Fe) evidences higher contents of this metal in the same biological matrix [26], namely, in the Brodmann area [21], frontal cortex [27], and amygdala [23]. Furthermore, epidemiological studies report an association between higher levels of Fe in soil and the diagnosis of AD [28] as well as between these concentrations and AD-related mortality [29]. Indeed, this metal seems to be responsible for oxidative stress and increases amyloid deposition [30]. Abnormalities of brain copper (Cu), iron (Fe) and zinc (Zn) homeostasis in AD were reported [31].

Systematic exposure to trace elements (TE) is a common occurrence worldwide. Human contamination may result from various pathways, namely, through inhalation of contaminated air/ambient particulate matter, ingestion of contaminated soil/dust, water and foodstuffs (such as agriculture crops, meat, and seafood), and dermal absorption of TE present in soil/dust [10,32,33]. The toxicity of each TE is dependent on the route and absorbed dose, which is in turn dependent on the concentration and duration of exposure [34]. Furthermore, each TE has a specific half-life, and the elimination of different elements from the body widely varies [35]. Previous studies report the significance of occupational exposure to specific TE and their effects on the development of cognitive disorders and dementia [5,6,35,36,37].

Despite the increasing evidence reporting the role of environmental exposures in the development of neurodegenerative diseases, the real effects of long-term exposure to TE in cognition remain unclear—literature on the possible pathogenic role of different metals do not provide direct evidence to define a causal relationship between environmental exposure to certain metals and AD [15,16]. Thus, the study of the influence of TE in the cognition of elderly people is necessary. The crossing of biological and neuropsychological assessment data to address this matter is an interesting approach. On the one hand, a comprehensive neuropsychological assessment is an essential component to identify subtle cognitive and behavioral deficits associated with neurotoxic exposure [38,39,40]. On the other hand, the use of fingernails for biomonitoring purposes provides a valid measurement of relatively long-term TE intake/exposure in a single specimen analysis [41]. Human nails are mainly constituted by keratin-rich proteins, and as they remain isolated from further metabolic activities after growth, they retain a high content of most TE. Moreover, the incorporation of TE in the nails’ matrix occurs in a time-integrated fashion and in proportion to the dietary intakes and other exposures to which an individual may be subjected [41,42]. Bioaccumulation ratios show that nails have higher mass/mass ratios (mass of TE per mass of sample) compared to blood and urine [32,41,43]. As nail samples are easy to collect (nail clipping is a non-invasive procedure), transport, and store [44], fingernails are useful and cost-effective specimens for element profiling.

Considering this translational approach, the present exploratory study comprises the cognitive assessment of a group of elderly individuals residing in potential environmentally-toxic geographic regions 5 years after the TE biomonitoring, in order to assess whether nail TE levels are associated with performance scores. The main aim is to investigate the effects of TE on future cognitive status.

## 2. Materials and Methods

### 2.1. Participants and Study Design

The present research comprised a study-group (RES) and a neuropsychological control-group (CTR). Criteria for participants’ inclusion accounted: (i) being a native Portuguese speaker, (ii) being over 50 years of age (given the likelihood of this cohort to report cognitive complaints and/or being involved in pathological aging processes of the dementia spectrum), (iii) having resided in the study areas for at least the past 5 years (prior to this study, in order to ensure minimum exposure time [45]; applicable for the RES group), (iv) having maintained the same occupation/professional activity.

The exclusion criteria were the following: (a) having a history of neurological disease (other than of the dementia spectrum); (b) having a history of psychiatric illness, including depression with the exception of stable mild depressive symptoms (depression symptomatology was assessed through the application of the Geriatric Depression Scale—30 [46,47,48]; individuals with total scores ≥ 21 were excluded); (c) having a significant visual, auditory, or language impairment that would negatively affect their ability to satisfactorily complete cognitive tests or understand test instructions; (d) current or prior alcohol, drugs, and other substances abuse; (e) current or prior use of antipsychotic medication; (f) being a smoker; (g) current or prior intake of supplements containing any of the analyzed TE; (h) significant changes in diet.

All subjects enrolled in the RES group were permanent residents from the municipality of Estarreja or from the municipality of Mértola and were voluntarily recruited through convenience sampling. Participants from the CTR group were randomly selected from an already existing longitudinal cohort composed of 250 community-residents recruited from several social institutions, aging associations and primary health care centers, who have been neuropsychologically assessed for the past 10 years. This cohort has been stratified according to several sociodemographic variables with a distribution similar to that observed in the Portuguese population, resulting in a cohort representative of the population of the country.

The RES group was initially composed of 76 participants who were cognitively assessed by an experienced neuropsychologist and whose fingernail clippings were collected and analyzed for biomonitoring of twenty selected TE: Al, arsenic (As), barium (Ba), cadmium (Cd), cobalt (Co), chromium (Cr), Cu, Fe, mercury (Hg), lithium (Li), manganese (Mn), nickel (Ni), lead (Pb), antimony (Sb), selenium (Se), tin (Sn), strontium (Sr), titanium (Ti), vanadium (V), and zinc (Zn). Out of the initial group, a total of 20 individuals were available for the second neuropsychological assessment, performed 5 years after the biomonitoring study. The neuropsychological data was paired with the biological data resulting from the nails TE profiling. In turn, the CTR group was composed of 20 subjects randomly selected from the mentioned pre-existing cohort of 250 subjects, previously matched by educational level with the RES group. For comparability reasons, since this pre-existing cohort has been followed for 10 years, the data considered for the CTR group referred to the 5-year follow-up benchmark.

All cognitive performances were assessed through the application of two cognitive screening tools: the Mini-Mental State Examination (MMSE [49,50]) and the Montreal Cognitive Assessment (MoCA [51,52]). The referred educational pairing between the two groups was performed based on previous evidences that report educational level as the variable that most significantly contributes to the prediction of scores on cognitive screening tests, such as MMSE and MoCA [53,54,55,56,57].

Ethical approval for the present research was obtained from the National Committee for Data Protection (No. 11726/2017). This study complies with the ethical guidelines for human experimentation stated in the Declaration of Helsinki committee and all participants gave written informed consent prior to participation after the aims and research procedures were fully explained by a member of the study group.

### 2.2. Geographical Areas of Interest

The study included residents from two distinct areas in Portugal—a northern industrial region (Estarreja) and a southern region associated with the mining industry (Mértola). Both areas display interesting characteristics to study the potential effect of long-term environmental exposure to chemicals.

Estarreja, a municipality located near one of the largest Portuguese cities, Aveiro, hosts one of the largest chemical complexes of the country. In this municipality, located near the small town with the same name, the Estarreja Chemical Complex (ECC) has been intensively operating since the 1950s in the production of aniline and derivatives, chlorine-alkalis, sodium and chlorate compounds through electrolysis using Hg cathodes, polyvinyl chloride resins, and polymeric methyl diphenyl isocyanate. In the past, the ECC also produced ammonium sulphate, ammonium nitrate, and sulphuric acid [58]. Until the 21st century such activity had serious deleterious effects on the environment with regards to soils/agricultural fields, surface waters, groundwater, and atmosphere [59,60,61,62,63]. Toxic solid wastes and liquid effluents were discharged directly into manmade, permeable water channels, without any previous treatment [58]. Environmental remediation works only began in 1998 [63].

Mértola is a municipality located in Baixo Alentejo, a southern area with several abandoned manganese mines spatially related with the Iberian Pyrite Belt. Mining releases large amounts of potentially toxic elements into the environment, which contaminate soils, surface waters, and groundwater. Right next to Mértola are the São Domingos Mines, the biggest Portuguese mining exploration until their shutdown in 1996. Close to the mines, the abandoned tailings deposits remain exposed to the weathering conditions that promote mineral leaching, with the release of elements such as As, Pb, Cd, and Cr. In addition, depending on the weather conditions, fine and ultrafine mineral particles are re-suspended and added to the ambient particulate matter, being transported to the neighboring areas [64,65,66].

### 2.3. Neuropsychological Assessment

All participants were inquired in regards of their sociodemographic features (i.e., age, educational level, nationality, number of years residing in the regions of interest, marital status) through a sociodemographic questionnaire; their current and past clinical history (i.e., weight, height, medical records) through a clinical structured interview; and their professional activity and consumption habits (i.e., main profession, working-time in agriculture/factories/mines, use of pesticides, consumption of home-grown foodstuff, and source of water for consumption/irrigation), through specifically developed questionnaires. Furthermore, all participants underwent a neuropsychological assessment performed by an experienced neuropsychologist. The screening tools of the assessment battery were administrated in a fixed order and were the following:

1. The Mini Mental State Examination (MMSE [49,50]), which is a brief cognitive screening tool widely used in the assessment of cognitive impairment [53]. It is composed of 30 dichotomous items (0—incorrect; 1—correct) and assesses 5 cognitive domains: Orientation, Memory, Attention and Calculus, Language, and Visuoconstruction. Higher scores indicate better cognitive performance.
2. The Montreal Cognitive Assessment (MoCA [51,52]), which is a screening tool developed to detect milder forms of cognitive decline, such as Mild Cognitive Impairment (MCI [67]). This instrument assesses 6 cognitive domains—Executive Functions; Visuospatial Abilities; Memory; Language; Attention Concentration and Working Memory; Temporal and Spatial Orientation—on a scale of 30 points, whose higher global scores translate better cognitive functioning.
3. The Geriatric Depression Scale—30 (GDS-30 [46,47,48]), which is a brief scale specifically developed for the screening of depressive symptoms in advanced adulthood. It is composed of 30 yes/no questions regarding the affective and cognitive domains of depression. Greater scores indicate more severe symptomatology.

### 2.4. Fingernail Samples and Analysis

Fingernail clippings from the RES participants were collected individually with a synthetic quartz knife and put into decontaminated plastic bags. Plastic forceps were used to remove visible exogenous material when needed.

The procedure used is described elsewhere [68]. Fingernail clippings were properly washed in order to remove exogenous contamination while preserving endogenous TE content. Samples were then dried at 95 °C in a laboratory drying oven (Raypa, Spain) until a constant weight was reached (ca. 40 h). The dried samples (approximately 0.1 mg) were mineralized in a Milestone (Italy) MLS 1200 Mega high-performance microwave digestion unit equipped with an HPR 1000/10 rotor, through a microwave-assisted acid digestion procedure using 1 mL of concentrated HNO_3_ (>69.0% m/m; TraceSELECT^®^, Fluka, France) and 0.5 mL of H_2_O_2_ (≥30% *v*/*v*; TraceSELECT^®^, Fluka, Germany). The used microwave oven program (W/min) was the following: 250/1, 0/2, 250/5, 400/5 and 600/5. After cooling, ultrapure water was added to the samples digest, and the volume was brought to 10 mL The solutions were then stored at 4 °C in closed propylene tubes until analysis.

All labware materials were decontaminated by immersion for at least 24 h in a 10% HNO_3_ bath followed by thorough rinsing with ultrapure water. The ultrapure water (resistivity > 18.2 MΩ·cm at 25 °C) was produced in an aarium^®^ pro (Sartorius, Germany) water purification system. For each digestion run (10 samples), a sample blank was prepared, and the average blank level was subtracted from the samples’ values. The certified reference material ERM-DB001—Trace Elements in Human Hair was used for analytical quality control, digested and analyzed through the same procedures as for the study samples. TE concentrations were determined through Inductively Coupled Plasma-Mass Spectrometry (ICP-MS) using a Thermo Fisher Scientific (Waltham, MA, USA) iCAP™ Q instrument equipped with a MicroMist™ nebulizer (Glass Expansion, Port Melbourne, Australia), a Peltier-cooled baffled cyclonic spray chamber, a standard quartz torch, and a two-cone interface design (nickel sample and skimmer cones) operated under the instrumental conditions presented in Table S1. High-purity argon (99.9997%; Gasin, Portugal) was used as nebulizer and plasma gas. The instrument was tuned before each analytical series for maximum sensitivity and signal stability and minimum formation of oxides and double-charged ions. Calibration standards and the internal standard solution were prepared from commercially available multi-element stock solutions (Plasma CAL, SCP Science, Baie-D’Urfe, QC, Canada, and ICP-MS Internal Std, Isostandards, Material, Madrid, Spain, respectively).

The following elemental isotopes were monitored for analytical determinations: ^7^Li, ^27^Al, ^48^Ti, ^51^V, ^52^Cr, ^55^Mn, ^57^Fe, ^59^Co, ^60^Ni, ^63^Cu, ^66^Zn, ^75^As, ^82^Se, ^8^8Sr, ^111^Cd, ^118^Sn, ^121^Sb, ^137^Ba, ^202^Hg, and ^208^Pb. The elemental isotopes ^45^Sc, ^89^Y, ^115^In, and ^159^Tb were used as internal standards. Limits of detection were calculated as the concentration corresponding to three times the standard deviation of 10 replicate measurements of the blank solution (2% *v*/*v* HNO_3_) and are presented in Table S2. Results for the certified reference material are presented in Table S3. The reference material used provides data for a limited set of TE, but the analytically most problematic elements are included. Since the analytical procedure has been validated for this set, accuracy problems are not expected for the other determined elements.

### 2.5. Statistical Analysis

Descriptive statistics were used to characterize both groups, RES and CTR, as well as the analytical results. Differences between the cognitive assessment scores of RES subjects at baseline and past five years were studied through a Wilcoxon signed-rank test analysis. Group differences between the cognitive decline registered for the RES group and for the CTR group were assessed through a Mann–Whitney U test. This option for non-parametric tests was based on the small size of the follow-up study group (the RES group). A probability of ≤0.05 was assumed as significant in testing the null hypotheses of no differences between the two moments of assessment and of no differences between the two groups. Potential relationships between TE contents in fingernails and neuropsychological data from the follow-up were firstly assessed through correlation analyses. Linear regression models (simple and multiple) were then computed to further investigate these relationships and assess whether TE significantly predicted future cognitive performance. A significance level of 95% was also used as criteria in both correlation and regression analyses. Comparisons between regression models were based on changes in the variance explained by each model, indexed by the adjusted coefficient of determination (R^2^_adjusted_). All statistical analyses were conducted using the Statistical Package for the Social Sciences (SPSS) for Windows, version 22 [69].

## 3. Results

### 3.1. Sample Characterization

The baseline and the follow-up assessments were separated by a 5-year interval (M = 5.30; SD = 0.47). Out of the 76 RES participants initially enrolled in the study, a total of 20 individuals (26.3%) could be included in the follow-up. Drop-outs were mainly due to death (42 out of 56 cases). Of the 42 deceases registered, 10 were due to cardiovascular disorders, 8 to respiratory infections, 8 to cancer, 5 to stroke, 4 to dementia complications (1 case of AD, 1 case of Parkinson’s disease, and 2 cases of non-specified dementia, diagnosed by general practitioners), 1 to gastrointestinal hemorrhage, and 6 due to unknown reasons. Other reasons for experimental death were medical conditions (2 cases), unreachability (9 cases), and unavailability (3 cases). Most RES participants enrolled in the follow-up were female (80%), with an average age of 83.60 ± 6.98 years, 2.45 ± 1.73 years of formal education (6 were illiterate), and a mean time of residence in the study areas of 61.93 ± 25.08 years. As for the education-matched control group (CTR), this was composed of 20 subjects, 55% of which were females, with a mean age of 74.70 ± 4.51 years and an average educational level of 3.30 ± 0.80 years (*p* > 0.05). Descriptive statistics of the two groups regarding marital status, occupation/professional activity, and medical history are presented in Table 1.

### 3.2. Neuropsychological Data

Overall, RES participants exhibited worse cognitive capacities at follow-up, comparatively to the baseline. Subjects scored on average less 6.5 points in MMSE (Baseline: M = 23.80; SD = 5.06; Follow-up: M = 17.30; SD = 6.94; *Z* = −3.628, *p* < 0.001, *r* = −0.811) and less 5.27 points in MoCA (Baseline: M = 16.00; SD = 4.472; follow-up: M = 10.73; SD = 5.985; *Z* = −2.317, *p* = 0.020, *r* = 0.699) after 5 years. When compared to the Portuguese normative data established according to age and educational level, the percentage of individuals classified as “with cognitive deficit” (i.e., with scores at 1 standard deviation or more below the expected mean considering age and education) raised from 65.0% to 85.0% according to the MMSE [70] and from 78.6% to 81.8% according to the MoCA [67]. The percentage of individuals with scores below the cut-off defined for AD, Frontotemporal Dementia (FTD), and Vascular Dementia (VD) raised from 60.0% to 85.0% according to the MMSE (*cut-off* of <26) [71,72,73] and from 71.4% to 83.3% according to the MoCA (*cut-off* of <17) [71,72,73].

In order to assess whether the cognitive decline of RES participants significantly differed from the cognitive decline observed in the general Portuguese population, differences between the RES group and the CTR group were explored based on the decline observed in MMSE and MoCA scores (decline = baseline score—follow-up score). While the CTR group exhibited a mean score difference of 1.40 ± 1.59 points in the MMSE and of 2.06 ± 1.35 points in the MoCA, the RES group presented score differences of 6.50 ± 4.27 and 5.27 ± 5.20 points, respectively. The observed decline was significantly different between the two groups for both the MMSE (U = 56.500, *p* < 0.001, *r* = −0.616) and the MoCA (U = 19.000, *p* = 0.005, *r* = −0.583).

### 3.3. TE Content in Fingernails

Descriptive statistics for TE content in fingernails of the RES group are summarized in Table 2. Observed values were compared to values reported in the literature for non-exposed people [74] and healthy centenarians [75]. For most TE, the obtained results were within the reported ranges, with Cd, Co, Li, and Pb contents falling in the lower part of the intervals reported for non-exposed individuals. The same was observed for Co and Mn contents when compared to the intervals reported for healthy centenarians. The contents of Ba, Sr, and Se fell within the reported range for non-exposed people but were below (Ba and Sr) and above (Se) the reference range for healthy centenarians.

### 3.4. Relationship between Fingernail TE Content and Cognitive Performance

In order to assess the relationship between TE in fingernails and the results of the neuropsychological assessment (total scores obtained with the MMSE and MoCA), a correlation analysis was performed. Correlation coefficients for the relationship between MMSE scores and Ni content (*r* = 0.489), MoCA scores and Ni content (*r* = 0.626), and MoCA scores and Se content (*r* = 0.647) were the only ones that reached statistical significance (*p* < 0.05), evidencing a moderate to strong positive association between these elements and the cognitive screening tests’ scores [76,77].

To further investigate whether TE contents could predict the cognitive performance of the RES group 5 years after the biomonitoring, linear regression analyses were run. The computed linear models assumed as predictor variables the TE contents of Ni and Se (the elements that led to significant correlation coefficients with the cognitive scores). Models are reported in Table 3.

Initially, simple linear regressions showed Ni as a significant predictor of MMSE total scores (β = 0.489, *t* = 2.381, *p* = 0.029), with the regression model (MMSE-1) explaining 19.7% of the variance of the scores. Regarding MoCA total scores, both Ni (β = 0.626, *t* = 2.538, *p* = 0.029) and Se (β = 0.647, *t* = 2.687, *p* = 0.023) showed as relevant predictors. After gathering both TE in a single regression model, the statistical significance for both variables persisted, with Se being highlighted as the best predictor variable (explaining 55.6% of MoCA scores, vs. the 52.9% for Ni). The resulting statistically significant model (MoCA-3; *p* = 0.005) could explain 62.2% of the MoCA total scores variance (an increase of 29.1% and 26.1% of the variance explained by the models MoCA-1 and -2, respectively).

Because education greatly influences cognitive performance on screening tests [53,54,55,56,57,78] and since aging seems to be associated with cognitive decline [63,64], age and educational level were included in the regression equations in order to account for these two confounding variables. Models for MMSE and MoCA with the inclusion of age showed a lack of statistical significance, and age was not a relevant predictor of the tests’ total scores (*p* > 0.05). Contrastingly, when the educational level was included into the equations, Ni and Se remained as relevant predictors, with the regression models showing statistical significance. Compared to the MMSE-1, a 9.1% increase in the variance explained by the model was observed, despite education being shown as a non-significant predictor (β = 0.358, *t* = 1.818, *p* > 0.05). In the resulting MMSE-2 model, both Ni and education explained together 28.8% of MMSE total scores. The same was true when the educational level was added to the MoCA-3 model. Although education was not a significant predictor (β = 0.304, *t* = 1.605, *p*> 0.05) of MoCA total scores, its inclusion caused an increase of 5.7% of the variance explained by the regression model. The resulting MoCA-4 model explained 67.9% of MoCA total scores. Therefore, MMSE-2 (where Ni: β = 0.556, *t* = 2.821, *p* = 0.012) and MoCA-4 (where Ni: β = 0.655, *t* = 3.441, *p* = 0.009; Se: β = 0.580, *t* = 3.329, *p* = 0.010) were selected as the final linear models. In MoCA-4 model, Ni is highlighted as the best predictor variable. In both MMSE-2 and MoCA-4 models, higher contents of Ni/Se in fingernails predict better cognitive performances in screening tests 5 years after biomonitoring analyses.

## 4. Discussion

Despite the increasing evidence reporting the role of environmental exposures in the development of neurodegenerative diseases, literature on the possible pathogenic role of different metals does not provide direct evidence to define a causal relationship between environmental exposure to certain metals and AD [15,16]. Therefore, research on the influence of TE on the cognition of elderly people is warranted. The present study aimed to explore potential relationships between TE levels in the human body and cognition. Specifically, the goal was to assess how TE content in fingernails relate to future cognitive performance.

Analyses of the RES neuropsychological data revealed an average decrease of 6.5 points and 5.27 points on the global scores of MMSE and MoCA, from baseline to follow-up (5 years later), as well as an increase in the portion of elderly individuals classified as “with cognitive deficit”—from 65.0% to 85.0% and from 78.6% to 81.8%, respectively. When compared to the CTR group, the decrease observed in MMSE and MoCA scores was significantly greater among the RES participants, indicating that the residents of the geographical areas of interest suffered a steeper cognitive decline than the general Portuguese population of the same educational level (within a 5-years period).

In terms of the RES participants with a cognitive performance similar to patients diagnosed with dementia (AD, FTD, and VD), they constituted 85.0% and 83.3% of the study group, according to the *cut-offs* of the screening tools [71,72,73]. These percentages of dementia-like performances are greater than expected considering the prevalence rate of the condition estimated for the Portuguese population (9.2% based on the 10/66 *Dementia Research Group*’ dementia diagnostic algorithm [79]), which indicates a higher frequency of cognitive impairment across the study group in comparison to the general Portuguese population older than 65 years. Literature systematically reports aging as the primary risk factor for neurodegenerative diseases, as this process implies a set of biological changes at the cellular level, from genomic instability to altered intercellular communication [80]. Indeed, cognitive decline seems to be intimately associated with age, and the prevalence of dementia cases increases exponentially after 65 years of age [80,81]. Considering that the average age of the RES elders included in this study was 83.6 years (with the youngest participant being 68 years old) and that the average age reported by Gonçalves-Pereira et al. for their study group was 74.9 years [79], it is plausible to admit that the difference observed in the frequency of cognitive impairment between both studies can be at least partially explained by the approximately 9-years gap between both groups of elders. However, the observed percentages of dementia-like performances seem to remain disproportioned even when compared to the prevalence estimated for the sub-group of individuals with ages above 80 years (18.37–19.61% based on the 10/66 *Dementia Research Group*’s dementia diagnostic algorithm [79]) assessed by Gonçalves-Pereira et al., suggesting that such differences are not attributed to age discrepancies.

The comparison between fingernails TE content in the RES group and values reported in the literature for non-exposed individuals [74] showed an overall good fit, suggestive of non-significant exposure to environmental contaminants. Nevertheless, it is worth noting that the mean age of the study sample (approximately 84 years) is substantially different from the average age of the population assessed by Rodushkin and Axelsson [74], which mean age was 33 years (range: 1–76 years).

With regards to the potential association between TE content in fingernails and cognitive performance, we found positive significant correlations between Ni and Se levels and the global scores of MMSE and MoCA. Regression analysis showed Ni and Se as significant predictors of future cognitive performances and revealed that, together with educational level, Ni explains 33% of the variance observed in MMSE total scores after 5 years, while Ni and Se explained 68% of the variance in MoCA. It is important to note that age proved to be a non-significant, non-relevant, predictor of the tests’ total scores, which is in line with the previous hypothesis that the cognitive decline observed among RES participants is not age-derived.

Nickel is a silver-white metal that belongs to the ferromagnetic elements group and that is widely distributed across the environment. It can be absorbed through the respiratory track, digestive system, and skin [82], and besides being essential for some plants and animal species [83], this metal is also a micronutrient essential for proper functioning of the human body (as it increases hormonal activity and is involved in lipid metabolism [82]). Depending on the duration, intensity, and pathway of exposure, Ni can act as an immunotoxin, as well as a carcinogenic agent, and cause several health problems (e.g., [84,85,86,87]). Furthermore, Ni accumulates in the brain and can act as a neurotoxicant [88]. Despite the molecular mechanisms not being well understood, its actions seem to be linked with oxidative stress, mitochondrial dysfunctions, and Ni-induced apoptosis (please see [83] and [88] for more details). Defective apoptotic processes may lead to excessive cell death, which underlies several neurodegenerative conditions. Toxicological studies in rats have shown alterations in the neuronal morphology of rat’s brain after Ni administration [89]. A significant reduction of intact neurons in the hippocampus and striatum was observed, as well as ultrastructural alterations in neurons of the hippocampus, striatum, and cortex [89,90]. Mitochondria seemed to be the key target in Ni-induced neurodegeneration [90]. Cognitive and motor behavior were also compromised [89].

Literature reports tend to portrait Ni as an element with neurotoxic repercussions that may affect cognition and contribute to the development of neurodegenerative diseases. Our study seems to contradict such evidence, as the results obtained showed that fingernail Ni content significantly predicts a lower cognitive decay within 5 years. However, the observation that Ni content in fingernails was within the interval reported for both non-exposed individuals [74] and healthy centenarians [75] is relevant, as it not only suggests that our study group was not significantly exposed to this TE but also that our results are limited to normal (non-toxic) content levels. Therefore, our findings suggest that, in spite of its potential neurotoxic effects, Ni may also have beneficial effects on long-term cognitive performance maintenance, provided it is maintained within certain concentration levels.

The role of Se in the human organism seems to be much better understood. Se exists in several chemical species [91,92,93], being a TE of interest for both nutritional and toxicological reasons. While it is an essential element for the biosynthesis of selenoproteins when in the selenocysteine-bound organic form [94], it is also a toxicant when in the selenomethionine and inorganic forms [93]. Furthermore, Se has a very narrow range of safe exposure [95], and both its deficiency and excess are associated with important adverse health effects [96]. In the human body, Se plays an essential antioxidant role [97] and is very important for the maintenance of the homeostasis of the central nervous system [98]. Its beneficial effects are mediated by selenium-containing proteins (the selenoproteins) such as glutathione peroxidases (GPx), the plasma Se-transport protein (SePP), and thioredoxin reductases (TrxR) [99]. One of the roles of these selenoproteins is to regulate oxidative stress, which has been linked with an increased risk of cognitive decline [100]. For GPX specifically, whose main function is to eliminate peroxides (therefore maintaining lower levels of reactive oxygen species), it is also known that is expressed in neurons and glia cells [101]. Additionally, Se also appear to be inversely associated with systemic inflammation, a critical process in age-associated cognitive decline and dementia [102,103]. In vitro studies observed this negative relationship in several cells, including neurons [104] and macrophages [105]. Such features are essential for neuroprotection and therefore brain metabolism of this TE is different from other organs [106]. In case of deficiency, the brain is the last organ to be depleted and is the first one to return to normal store levels when Se is replenished [107].

Given its beneficial effects, Se has been widely studied within the scope of healthy aging and cognitive impairment. Reduced levels of Se are believed to lead to neurons destruction and increased risk of cognitive decline/dementia [108,109]. Studies show that, compared to healthy elder adults, AD patients exhibited reduced levels of Se in plasma, erythrocyte, blood, cerebrospinal fluid (CSF), and nails [97,110,111,112]. Specifically, the levels of this TE seem to be decreased in the temporal, hippocampal, and cortex regions of AD patients [113]. Higher CSF levels of selenite (a common inorganic Se species) seem to predict progression to AD in non-vascular MCI patients [114]. Studies on Chinese elderly report an association between lower levels of Se in nails and poorer cognitive performances and that apoE4 carriers have significantly lower nail content of Se than non-carriers [111,113]. Concordantly to this evidence, our findings highlight the beneficial effects of Se in cognition, as higher fingernail Se contents predict lesser cognitive decline in 5 years. The determined Se content in fingernails was also within the reported interval for non-exposed individual [74], which suggests non-excessive exposure and lines up with the well-known U-shaped relationship between Se levels in the human body and adverse health effects. Interestingly, Se levels were higher in our participants when compared with healthy centenarians [75]. As age increases, the decrease of organs’ physiological functioning can greatly affect the absorption, distribution, metabolism, and function of Se in the human organism [115]. Given the differences in age between our study group (with a mean age of approximately 84 years) and the centenarians, this may explain the difference in Se levels. Indeed, previous studies report a reduction of Se levels in red blood cells not only in AD and MCI but also in normal elderly people [116,117]. Nevertheless, information on this topic is inconsistent, as studies have also shown higher levels of Se in the plasma among centenarians, when compared to septuagenarians [118] and nonagenarians [119]. An important variable contributing for the heterogeneity of results is the choice of different tissues and fluids when assessing TE levels. Literature findings show how the relationship between Se and certain diseases may vary across studies based on different tissues [120].

The main limitation of the present research lies on the number of drop-outs, mainly due to participants passing away (55% of the initial study sample), which led to a reduced sample size that potentially may have prevented results from reaching statistical significance. Notwithstanding, this limitation stems from a longitudinal methodology based on a 5-years follow-up that was implemented in a cohort whose average age is higher than the life expectancy of its population (estimated to be 80.8 years for Portugal [121]). Furthermore, the present study does not include the measurement of TE in the CTR group, and therefore, results should be interpreted in a preliminary/exploratory framework.

## 5. Conclusions

The present exploratory study provides original preliminary evidence of an existing association between the concentration levels of TE in the human body and future cognitive status—our findings show that fingernail contents of Ni and Se predict lesser cognitive decline within 5 years. Future expansion of the present research is warranted. More comprehensive samples and individual-level measurements of TE for control groups must be included in order to assess the real beneficial potential of these TE in the maintenance of cognitive capacities. Further research on humans is needed, and similar translational approaches based on crossing other biomarkers with neuropsychological data should be considered. Such contributions have the potential to open new avenues for a more successful prevention of cognitive impairment and dementia.

## Figures and Tables

**Table 1:** Descriptive statistics of the total sample in terms of marital status, occupation/professional activity, and medical history, discriminated by group.

	RES *n* = 20	CTR *n* = 20
**Marital Status, *n* (%)**		
Single	2 (10)	1 (5)
Married	2 (10)	12 (60)
Divorced	3 (15)	1 (5)
Widowed	13 (65)	6 (30)
**Occupation/professional activity, *n* (%)**		
Agriculture/fishery	6 (30)	1 (5)
Industry/construction	4 (20)	3 (15)
Commerce/Services	8 (40)	14 (70)
Housewife	2 (10)	3 (10)
**Medical History, *n* (%)**		
Diabetes	4 (20)	2 (10)
Dyslipidemia	12 (60)	9 (45)
Cardiovascular diseases	16 (80)	15 (75)

**Table 2:** Descriptive statistics for the observed trace elements (TE) contents in fingernails (research study-group (RES)), expressed as µg/g; values reported in the literature are presented for comparison purposes.

	RES Group (µg/g)								Literature Data			
					Non-Exposed Individuals [74]				Healthy Centenarians [75]			
	Min-Max	*M*	*SD*	*Md*	Min-Max	*M*	*SD*	*Md*	Min-Max	*M*	*SD*	*Md*
Al	3.57–54.61	18.93	14.22	14.42	12.00–137.00	36.00	22.00	32.00	-	-	-	-
As	0.07–0.30	0.12	0.06	0.11	0.07–1.09	0.27	0.19	0.22	-	-	-	-
Ba	0.11–2.21	0.64	0.73	0.31	0.28–3.99	1.34	1.35	0.89	0.94–22.92	5.10	3.92	3.85
Cd	0.002–0.06	0.01	0.01	0.01	0.01–0.44	0.11	0.18	0.06	0.004–0.19	0.03	0.03	0.02
Co	0.003–0.04	0.01	0.01	0.01	0.01–0.12	0.03	0.03	0.02	0.01–0.64	0.10	0.10	0.07
Cr	0.25–0.93	0.61	0.20	0.60	0.22–3.20	1.16	1.05	0.76	0.08–2.51	0.82	0.44	0.82
Cu	2.66–7.40	4.67	1.32	4.83	4.20–17.00	8.40	3.50	7.60	2.02–8.53	3.71	0.99	3.55
Fe	7.31–49.84	24.01	13.25	20.06	12.00–189.00	42.00	30.00	37.00	16.52–692.00	154.40	124.80	116.70
Hg	0.12–0.85	0.42	0.20	0.42	0.03–0.31	0.12	0.098	0.098	-	-	-	-
Li	0.005–0.18	0.04	0.04	0.02	0.01–0.25	0.07	0.07	0.05	0.02–2.07	0.31	0.32	0.23
Mn	0.04–2.93	0.58	0.79	0.17	0.19–3.30	0.90	0.75	0.65	0.21–15.40	3.09	2.18	2.62
Ni	0.09–2.98	0.93	1.04	0.36	0.14–6.95	1.65	2.20	0.84	0.02–3.67	0.95	0.85	0.66
Pb	0.07–1.73	0.38	0.38	0.30	0.27–4.75	1.38	1.14	1.06	0.13–9.61	1.86	1.81	1.33
Sb	0.008–0.13	0.04	0.04	0.03	0.01–0.13	0.05	0.05	0.04	-	-	-	-
Se	0.60–1.03	0.803	0.13	0.80	0.62–1.53	0.94	0.21	0.93	0.24–0.70	0.44	0.11	0.44
Sn	0.01–1.20	0.33	0.36	0.17	0.11–2.56	0.63	0.51	0.48	-	-	-	-
Sr	0.07–2.24	0.48	0.53	0.27	0.17–1.39	0.43	0.21	0.39	1.40–18.50	6.20	2.47	5.80
Ti	3.68–6.75	5.14	0.77	5.15	0.94–16.10	4.46	5.01	2.71	-	-	-	-
V	0.05–0.38	0.120	0.07	0.10	0.02–0.48	0.08	0.05	2.71	-	-	-	-
Zn	88.65–219.31	144.10	39.15	137.34	80.00–191.00	120.00	29.00	116.00	93.00–326.00	148.00	36.00	138.00

Note: Min = minimum; Max = maximum; *M* = mean; *SD* = standard deviation; *Md* = median; Al = aluminum; As = arsenic; Ba = barium; Cd = cadmium; Co = cobalt; Cr = chromium; Cu = copper; Fe = iron; Hg = mercury; Li = lithium; Mn = manganese; Ni = nickel; Pb = lead; Sb = antimony; Se = selenium; Sn = tin; Sr = strontium; Ti = titanium; V = vanadium; Zn = zinc.

**Table 3:** Linear regression models.

	Linear Regression Models	R^2^_adjusted_	Model Significance
**MMSE-1**	MMSE = 14.258 + 3.259 Ni	0.197	*p* = 0.029
**MMSE-2**	MMSE = 10.329 + 1.436 Education + 3.700 Ni	0.288	*p* = 0.022
**MoCA-1**	MoCA = 7.503 + 3.369 Ni	0.331	*p* = 0.029
**MoCA-2**	MoCA = −9.258 + 26.821 Se	0.361	*p* = 0.023
**MoCA-3**	MoCA = −9.460 + 2.851 Ni + 23.017 Se	0.622	*p* = 0.005
**MoCA-4**	MoCA = −16.147 + 1.556 Education + 3.525 Ni + 24.022 Se	0.679	*p* = 0.007

Note: MMSE = Mini Mental State Examination; MoCA = Montreal Cognitive Assessment; MMSE-n = regression models assuming MMSE total scores as the dependent variable; MoCA-n = regression models assuming MoCA total scores as the dependent variable.

## Supplementary Materials

The following are available online at https://www.mdpi.com/1660-4601/17/17/6051/s1. Table S1: Operating conditions for the iCAP^TM^Q ICP-MS instrument. Table S2: Limits of detection (LOD) for the analyzed trace elements. Table S3: Results obtained in the analysis of the certified reference material ERM^®^—DB001 Trace Element in Human Hair.
