# Supplementary material for: Associations between Trace Elements and Cognitive Decline: An Exploratory 5-Year Follow-Up Study of an Elderly Cohort
Source: Int J Environ Res Public Health. 2020 Aug 20;17(17):6051. doi: 10.3390/ijerph17176051 (PMC7503463; doi:10.3390/ijerph17176051)
Supplement: Supplementary file 1 [file ijerph-17-06051-s001.pdf]

Supplementary Materials

Table S1. Operating conditions for the iCAP™ Q ICP-MS instrument.

Plasma gas flow rate	14 L/min
Auxiliary gas flow rate	0.85 L/min
Nebulizer gas flow rate	1.02 L/min
RF power	1550 W
Nebulizer	MicroMist™
Spray chamber	Baffled quartz cyclonic
Spray chamber temperature	2.7 ºC
Dwell time	20 ms*
Replicates	3
Sample uptake time	1 min
Washout time	2 min

* A dwell time of 50 ms was used for Hg.

Table S2. Limits of detection (LOD) for the analyzed trace elements.

Element	LOD (µg/g)
Al	0.047
As	0.032
Ba	0.002
Cd	0.001
Co	0.001
Cr	0.005
Cu	0.006
Fe	0.544
Hg	0.022
Li	0.001
Mn	0.006
Ni	0.004
Pb	0.001
Sb	0.002
Se	0.139
Sr	0.004
Ti	0.010
V	0.004
Zn	0.013

Table S3. Results obtained in the analysis of the certified reference material ERM® – DB001 Trace Element in Human Hair.

Element	Certified value ± uncertainty (μg/g)	Determined value ± standard deviation (μg/g)*	Recovery (%)
As	0.044 ± 0.006	0.053 ± 0.006	121.0 ± 14.3
Cd	0.125 ± 0.007	0.114 ± 0.003	91.0 ± 2.2
Cu	33.000 ± 4.000	31.400 ± 0.600	95.1 ± 1.8
Hg	0.365 ± 0.028	0.329 ± 0.020	90.2 ± 5.5
Pb	2.140 ± 0.200	2.260 ± 0.080	105.8 ± 3.9
Se	3.240 ± 0.240	3.210 ± 0.520	99.2 ± 8.9
Zn	209.000 ± 12.000	198.000 ± 2.400	94.6 ± 1.1

* Data from two independent digestions of the certified reference material with triplicate analysis of the sample solutions (at the beginning, middle, and end of the analytical run).
